# Supplementary material for: Prognostic factors in severe dengue patients: A multi-center retrospective cohort study
Source: PLoS Negl Trop Dis. 2025 Jan 28;19(1):e0012846. doi: 10.1371/journal.pntd.0012846 (PMC11805397; doi:10.1371/journal.pntd.0012846)
Supplement: Attachment — Submitted filename: List of revision.docx [file pntd.0012846.s004.docx]

<< List of revision >>

**Reviewer #2:** Which normality test was used before applying the Chi-square test or Fisher's exact test, the Student t test, or the Mann-Whitney U test? Please include this information to ensure the correct application of the tests.

Response:

Thank you for the reviewer’s valuable comments. In our research, we used the Chi-square test or Fisher's exact test for categorical variables and the Mann-Whitney U test for numerical variables. A normality test is not required for these tests because they are applied to categorical data and do not assume a normal distribution. Additionally, the Mann-Whitney U test is a non-parametric test, meaning it can be used for data that does not follow a normal distribution. In the revised version, we have added the following clarification: “The Chi-square test or Fisher's exact test was used for categorical variables, while the Mann-Whitney U test was applied to numerical variables. A normality test is not required for categorical data when using the Chi-square test or Fisher's exact test. As the Mann-Whitney U test is non-parametric, it also does not require a normality test”. (lines 174-177).

**Reviewer #2:** I understand that the results present many variables, which may make it difficult to display these data in a single graph. However, I still recommend creating graphs for at least the comparisons of the statistically significant variables. You could create a graph for each type of variable, for example: Graph A for the statistically significant data from the variable "Symptoms and signs upon arrival," Graph B for "Laboratory results upon arrival," and so on. This way, the graphical presentation would not duplicate the extensive information in the tables but would highlight the "most important" ones, making it clearer for the reader and further improving the overall quality of the article.

Response:

Thank you for the reviewer’s valuable comments. In the revised version, we have added Figures 1 and 2. Figure 1 illustrates the significant symptoms and signs at hospital presentation in survivors versus non-survivors, while Figure 2 presents the significant laboratory findings at hospital presentation and throughout hospitalization in survivors versus non-survivors (lines 215, 217, 218, 222, and 528-537).

--------------------

Reviewer #2: (No Response)

--------------------

Reviewer #2: (No Response)

--------------------

**Reviewer #2:** The authors have made the modifications and included the points I suggested in the first review. The article is now more robust and reliable. I believe that including the statistical test used to verify the normal distribution of the data and attempting to create the suggested graphs would significantly improve the article.

Response:

In the revised version, the statistical test was verified (lines 174-177), and two figures were added, depicting the significant symptoms/signs and laboratory findings between survivors and non-survivors (lines 215, 217, 218, 222, and 528-537).
