# Supplementary material for: Prognostic factors in severe dengue patients: A multi-center retrospective cohort study
Source: PLoS Negl Trop Dis. 2025 Jan 28;19(1):e0012846. doi: 10.1371/journal.pntd.0012846 (PMC11805397; doi:10.1371/journal.pntd.0012846)
Supplement: Attachment — Submitted filename: Response to reviewer (R3).docx [file pntd.0012846.s005.docx]

**List of revision**

PNTD-D-23-01029R2

Prognostic factors in severe dengue patients: A multi-center retrospective cohort study

Additional Editor Comments:

Your manuscript received one additional review. The review is an statistical concern that should be addressed before considering a revised version of your manuscript.

Response:

In the revised version, we included the following statement: "The normality of data variables was assessed using the Kolmogorov–Smirnov test, which identified hematocrit as the only variable following a normal distribution. Continuous variables were analyzed using the Student's t-test or the Mann–Whitney U test, with results presented as median and range. Categorical variables were compared using the χ² test or Fisher's exact test and are reported as frequency and percentage" (lines 174-178).

Journal Requirements:

1) We have noticed that you have uploaded Supporting Information files, but you have not included a list of legends. Please add a full list of legends for your Supporting Information files after the references list.

Response:

The list of legends for Supporting Information has been added to the text following the references section (lines 552-555).

Reviewer #2: The authors stated that “Additionally, the Mann-Whitney U test is a non-parametric test, meaning it can be used for data that does not follow a normal distribution” and “As the Mann-Whitney U test is non-parametric, it also does not require a normality test.” This is an absolutely incorrect statement. To determine if data follows a normal distribution, a normality test must be performed. It is not up to the author to decide whether the data has a normal distribution or not. The normality test should be conducted to verify this, after which one can select whether to use a parametric or a non-parametric test. Also, a non-parametric test does not imply that data lacks a normal distribution; rather, if data does not follow a normal distribution, a non-parametric test is used.

The authors should conduct a normality test to verify if the data follows a normal distribution and include this information for verification.

Response:

Thank you for the reviewer’s valuable comment. In the revised version, we have added the following statement: “The normality of data variables was assessed using the Kolmogorov–Smirnov test, which identified hematocrit as the only variable following a normal distribution. Continuous variables were analyzed using the Student's t-test or the Mann–Whitney U test, with results presented as median and range. Categorical variables were compared using the χ² test or Fisher's exact test and are reported as frequency and percentage” (lines 174-178).
