# Supplementary material for: Prognostic factors in severe dengue patients: A multi-center retrospective cohort study
Source: PLoS Negl Trop Dis. 2025 Jan 28;19(1):e0012846. doi: 10.1371/journal.pntd.0012846 (PMC11805397; doi:10.1371/journal.pntd.0012846)
Supplement: Attachment — Submitted filename: reviewer comment.docx [file pntd.0012846.s003.docx]

**Reviewer #1:**

This is an interesting study because it is a disease with a major impact on public health and without a vaccine widely used in endemic countries. However, some aspects need to be improved before this manuscript is accepted for publication, which I will list below.

1) Improvement in the definition of inclusion and exclusion criteria: it is not clear whether only critically ill patients were included or whether patients with warning signs were included "The warning signs, as defined by the 2009 WHO, include abdominal pain or tenderness, persistent vomiting, clinical fluid accumulation, mucosal bleeding, and lethargy/restlessness. However, we were unable to include hepatomegaly and an increase in hematocrit concurrent with a rapid decrease in platelet count as warning signs in our analysis due to the lack of information. SD was defined based on the 2009 WHO criteria, which categorizes it as dengue"

Response:

Thanks for the reviewer’s valuable comment. In the revised version, the inclusion and exclusion criteria were described as follows (lines 126-128): "The inclusion criteria for this study were dengue patients who exhibited severe dengue (SD), as defined by the 2009 WHO criteria [8]. Patients with non-severe dengue, both with and without warning signs, were excluded from the study". Further, we have added “Additionally, the warning signs outlined by the WHO in 2009 that manifested in severe dengue (SD) patients were recorded for analysis” (lines 133-134) in the revised version.

2) Characterization of the patient sample: How were they selected? Are these hospitals reference hospitals, but do they also serve patients through spontaneous demand?

Response:

Thanks for reviewer’s comment. In the revised version, we have added “We conducted a retrospective study that included eligible patients aged 18 years and older with laboratory-confirmed dengue between 2002 and 2019. These patients received treatment at Kaohsiung Chang Gung Memorial Hospital (2,700 beds) and Kaohsiung Medical University Hospital (1,700 beds), both of which serve as primary care and tertiary referral centers in Taiwan. These hospitals provide comprehensive services, including outpatient and inpatient care, as well as a 24-hour emergency department for patients requiring immediate medical attention” (lines 120-126).

**Reviewer #2:**

The objectives of the study are clearly articulated, and the study design is appropriate to address the stated objectives. However, I suggest the inclusion of some missing information to ensure the reliability of the data and reproducibility.

Patients and Setting:

1) The authors described the criteria to confirm the dengue cases included in the study. I suggest adding a brief explanation of how each of the assays was conducted and/or providing a reference.

-Additionally, the primer sequences used in the RT-PCR should be included, along with the cycling parameters of PCR, the controls of the reaction, the machine used for running, and the criteria to consider a sample as positive (e.g., ct value).

-Was the detection of IgM and IgG performed using a kit? If so, how was this detection performed? Please clarify these methodologies, and if applicable, add the kit or reagents used.

Response:

Thank you for the reviewer's comments. In this study, we employed the SD DENGUE IgM/IgG CAPTURE enzyme-linked immunosorbent assay for the detection of IgM/IgG. Additionally, the dengue virus-specific nonstructural glycoprotein-1 antigen assay was performed using kits from Bio-Rad Laboratories (Marnes-la-Coquette, France), AsiaGen Corp (Taiwan), and SD BIOLINE (Korea). For real-time reverse transcription polymerase chain reaction (RT-PCR), the QuantiTect SYBR Green RT-PCR kit (Qiagen, Hilden, Germany) was used. Primer sequences for detecting and serotyping the dengue virus are provided in the text, along with the detection limits for the four dengue virus serotypes. Additionally, three references (ref 13-15) were added for further information. These details are listed between lines 135 and 153 in the revised manuscript.

Definitions:

1) The authors stated that "severe hepatitis was defined as serum alanine aminotransferase (ALT) levels greater than 1,000 U/L (reference value, <40 U/L)." It would be clearer if the authors added why this cutoff of ALT levels was considered and provided a reference for it.

Response:

Thank you for the reviewer's comment. Severe hepatitis, defined as a serum alanine aminotransferase (ALT) level greater than 1,000 U/L, aligns with the definition of severe dengue according to the 2009 WHO dengue criteria (lines 163-166). Additionally, a reference (ref 16) concerning acute liver failure has been added.

**Reviewer #2:**

The analysis presented matches the analysis plan. However, the presentation of results could be improved.

1) I suggest presenting the results comparing survivors and non-survivors in a histogram graph. This approach would provide a more visual representation and assist readers in discerning the differences between the groups, along with the accompanying statistical analysis.

Response:

Thank you for the reviewer's appreciative and valuable comments. The table includes extensive information such as demographics, symptoms/signs, warning signs, laboratory data upon arrival and during hospitalization, and complications, featuring both categorical and numerical data types. Consequently, a histogram graph was not employed for the analysis between survivors and non-survivors. To aid readers in more comprehensively understanding and accessing key results, statistically significant variables are highlighted in bold within the table.

2) Additionally, if the data regarding the characteristics of fatal patients is presented in table 2, it should be referenced in the text. Therefore, Table 2 should be addressed in the text accordingly.

Response:

Thank you for the reviewer's comment. The independent risk factors for fatal outcomes in severe dengue patients have been addressed in the Results section of the revised manuscript (lines 223-231).

**Reviewer #2:**

The discussion and conclusions effectively support the presented data, addressing the public health relevance and discussing how the data can be beneficial. Additionally, the study limitations are appropriately addressed. However, to ensure robustness, please add references for the following sentences:

1) “These immunological changes further contribute to the risk of developing SD and mortality in this aging population.”

Response:

Thank you for your comment. In the revised manuscript, we have included Reference 25: Rowe EK, Leo YS, Wong JGX, Thein TL, Gan VC, Lee LK, et al. Challenges in Dengue Fever in the Elderly: Atypical Presentation and Risk of Severe Dengue and Hospita-Acquired Infection. PLoS Negl Trop Dis. 2014; 8: e2777.

2) “The WHO has documented that mucosal bleeding and the presence of lethargy or restlessness are pivotal warning signs for the development of SD”

Response:

In the revised manuscript, we have included Reference 8: Dengue: Guidelines for Diagnosis, Treatment, Prevention and Control: New Edition. Geneva: World Health Organization; 2009. PMID: 23762963.

3) “Moreover, severe plasma leakage in SD may lead to reduced cerebral perfusion, compromising the proper functioning of the brain, especially in susceptible populations such as elderly patients with multiple comorbidities.”

Response:

In the revised manuscript, we have included Reference 29: Salomão N, Rabelo K, Basílio-de-Oliveira C, Basílio-de-Oliveira R, Geraldo L, Lima F, et al. Fatal Dengue Cases Reveal Brain Injury and Viral Replication in Brain-Resident Cells Associated with the Local Production of Pro-Inflammatory Mediators. Viruses. 2020;12:603.

4) “Gastrointestinal bleeding can lead to hypovolemia, circulatory collapse, and shock. Delayed and inadequate fluid replacement, as well as insufficient transfusion of blood products in cases of severe bleeding, contribute to the development of circulatory failure and increased mortality.”

Response:

In the revised manuscript, we have included Reference 32: Huang WC, Lee IK, Chen YC, Tsai CY, Liu JW. Characteristics and predictors for gastrointestinal hemorrhage among adult patients with dengue virus infection: Emphasizing the impact of existing comorbid disease(s). PLoS One. 2018;13:e0192919.

5) “The presence of organ impairment during hospitalization is another crucial factor contributing to mortality in patients with SD”

Response:

In the revised manuscript, we have included Reference 13: Lee IK, Lee NY, Huang WC, Hsu JC, Tai CH, Yang CH, et al. In-hospital mortality predictors among hospitalized adults and those with chronic kidney disease with dengue. J Microbiol Immunol Infect. 2023;56:996-1006; and Reference 34: Rivera J, Rengifo AC, Alvarez-Díaz D, Parra E, Usme-Ciro J, Castellanos J, et al. Multisystem Failure in Fatal Dengue: Associations between the Infectious Viral Serotype and Clinical and Histopathological Findings. Am J Trop Med Hyg. 2023;109:908-916.

60 “Prolonged hypotension and shock resulting from severe plasma leakage and/or bacterial co-infection in cases of SD could lead to organ impairment such as liver and kidney damage. Moreover, the development of pneumonia indicates nosocomial infection, which can exacerbate the dengue disease and contribute to fatal outcomes.”

Response:

In the revised manuscript, we have included Reference 41: Thein TL, Ng EL, Yeang MS, Leo YS, Lye DC. Risk factors for concurrent bacteremia in adult patients with dengue. J Microbiol Immunol Infect. 2017;50:314-320; and Reference 42: Mehmood A, Afzal MW, Ahmad M, Mufti M, Malik J, Zaidi SMJ. Respiratory sequelae of dengue fever. Trop Doct. 2023;53:237-240.

**Reviewer #2:**

1) The introduction lacks essential information to contextualize the methods used and the obtained results. It would be beneficial to enrich the introduction to provide more context. For example, the authors could address concepts in the introduction that were not discussed but are pertinent to the results, such as "Dengue serotype 2." I suggest adding a section to the introduction describing dengue serotypes.

Response:

Thank you for your comment. We have updated the Introduction section of the revised manuscript to include: "Dengue is caused by four genetically related but antigenically distinct serotypes of the dengue virus (DENV-1, DENV-2, DENV-3, and DENV-4) [4]. Infection provides life-long immunity to the homologous serotype but only transient protection against the other three serotypes [5]. Secondary dengue infection significantly increases the risk of severe clinical outcomes such as dengue hemorrhagic fever and dengue shock syndrome, primarily due to antibody-dependent enhancement. [6]. In Taiwan, all four serotypes have been detected, with DENV-1 and DENV-2 being predominant, especially during the 2014-2015 epidemic [7]" (lines 84-91). Four references (ref 4-7) were added in the revised version.

Additionally, we added, " Factors such as secondary dengue infections, age, viral load, and the infecting serotype and genotype have been implicated in the progression of SD [9-11]. Notably, the outbreak of DENV-2 in Brazil was associated with heightened disease severity and a significantly elevated mortality rate [12]" (lines 98-101). Four references (ref 9-12) were added in the revised version.

2) Additionally, the authors should verify and correct the writing of the virus name throughout the text.

Response:

Thanks for reviewer’s comment. We have verified the term 'DENV' throughout the text.

Additional Amendments

1. The author summary has been rewritten (lines 60-73).
2. The Financial Disclosure statement has been updated (lines 372-375).
